# Acute Effects of Static Stretching Combined with Vibration and Nonvibration Foam Rolling on the Cardiovascular Responses and Functional Fitness of Older Women with Prehypertension

**DOI:** 10.3390/biology11071025

**Published:** 2022-07-07

**Authors:** Che-Hsiu Chen, Chin-Hsien Hsu, Lee-Ping Chu, Chih-Hui Chiu, Wen-Chieh Yang, Kai-Wei Yu, Xin Ye

**Affiliations:** 1 Department of Sport Performance, National Taiwan University of Sport, Taichung 404401, Taiwan; jakic1114@ntus.edu.tw; 2 Department of Leisure Industry Management, National Chin-Yi University of Technology, Taichung 41170, Taiwan; hsu6292000@yahoo.com.tw (C.-H.H.); kaiwei40150@gmail.com (K.-W.Y.); 3 Department of Orthopedics, China Medical University Hospital, Taichung 404332, Taiwan; d30886@mail.cmuh.org.tw; 4 Department of Exercise Health Science, National Taiwan University of Sport, Taichung 404401, Taiwan; chiuch@ntus.edu.tw; 5 Department of Physical Therapy, Hung Kuang University, Taichung 433304, Taiwan; wcyang@sunrise.hk.edu.tw; 6 Department of Rehabilitation Sciences, University of Hartford, West Hartford, CT 06117, USA

**Keywords:** aging, cardiovascular risk, flexibility, strength, balance

## Abstract

**Simple Summary:**

Thirty-seven percent of the US adult population have prehypertension, and a quarter to half of these over 65 years of age progress to hypertension in four years. Along with healthy diet, exercise or physical activity is one of the critical lifestyle factors for this population. General exercise recommendation or prescription to individuals who have cardiovascular risks is provided by organizations such as ACSM and AHA, but more detailed information and research are still needed. As the first component of any exercise program, finding the proper warm-up routine is important. We aimed to examine the acute immediate effects of three different warm-up protocols on cardiovascular responses and functional fitness testing in older women with prehypertension. Thirteen qualified subjects went through three protocols (static stretching with and without foam rolling, and stretching with vibration rolling) in three different sessions. Blood pressure was not altered only in the static stretching with foam rolling condition. Interestingly, adding the vibration component to the stretching increased the upper body flexibility and stretching. We therefore suggest the combination of static stretching with foam rolling as the safe and effective protocol for older women with prehypertension.

**Abstract:**

We compared the effects of three warm-up protocols (static stretching (SS), static stretching with vibration foam rolling (SS + VFR), and static stretching with nonvibration foam rolling (SS + FR) on the blood pressure and functional fitness performance in older women with prehypertension. Thirteen older women went through different protocols in separate visits, and their systolic (SBP) and diastolic (DBP) blood pressure, heart rate, mean arterial pressure, brachial pulse pressure (BPP), functional fitness test (back scratch (BS), chair-sit-and-reach, 30 s arm curl (AC), 30 s chair stand, 2 min step, 8-foot up and go), and single-leg standing balance (SLB) were recorded. The SBP and BPP were significantly higher after SS and SS + VFR than after SS + FR. Both SS + FR and SS + VFR significantly improved the 2 min step, when compared with SS. Additionally, SS + VFR significantly improved the BS and AC performance. However, compared with SS and SS + FR, SS + VFR significantly reduced the SLB performance. Therefore, SS + FR may be a better warm-up protocol for older women in maintaining blood pressure. On the other hand, even though SS + VFR induced superior shoulder flexibility, aerobic endurance, and arm strength, it could impair balance.

## 1. Introduction

Aging is generally associated with the degeneration of functional fitness, acceleration of structural and functional changes to the arterial wall, and increased vascular stiffness and endothelial cell dysfunction, resulting in increased blood pressure, including systolic blood pressure (SBP), diastolic blood pressure (DBP), mean arterial pressure (MAP), and pulse pressure (PP) [1,2,3]. Prehypertension (SBP: 120–139 mmHg or DBP: 80–89 mmHg) increases the risks of cardiovascular disease, coronary heart disease (CHD), and stroke [4]. Furthermore, middle-aged and older women have a higher prevalence of hypertension and greater aortic stiffness compared with their male counterparts [5]. As a frequent precursor of hypertension, 26–50% of the prehypertensive individuals ≥65 years of age progress to hypertension in four years [6]. Physical inactivity serves as one of the lifestyle factors to this progression [7]. Thus, exercise or physical activity is especially important for this population [8]. As the first component of any exercise program, finding the proper warm-up routine (e.g., preparing the individual to do exercise while not putting too much stress to the cardiovascular system, which can lead to complications) can be critical.

Static stretching (SS) is often used as a daily or warm-up exercise, which can enhance flexibility [9]. By contrast, decreases in muscle strength and sports performance have been reported after SS [10]. Recent studies have also suggested that SS improves vascular stiffness and endothelial function [11,12]. For instance, the femoral–ankle and brachial–ankle pulse-wave velocity gradually declined in healthy young men performing a single session of SS (involving the trunk, upper limbs, and lower limbs) for 40 min [12]. Additionally, a single session of passive calf stretching (six repetitions of 30 s SS) significantly reduced the arterial diameter during SS, but the mean blood velocity and shear rate increased during relaxation, which may affect nitric oxide production in endothelial cells and the attenuation of local arterial stiffness [11,13]. However, SS can also increase heart rate (HR), SBP, DBP, and the rate-pressure product [12,14,15,16], while reducing oxygen saturation (SpO_2_) and suppressing the vagal drive to the heart [17] in healthy young adults and adults, suggesting that SS increases cardiac overload.

Foam rolling (FR) [18,19] or vibration foam rolling (VFR) [20] is a relatively simple technique that has become increasingly popular and has been used as a component of warm-up exercises. Studies showed that FR can reduce sympathetic activity, SBP, DBP, and arterial stiffness acutely [21,22] and improves vascular endothelial function in healthy young individuals [21,23]. Although there is very limited information regarding local VFR on cardiovascular responses, one study reported that whole-body vibration (three sessions of 1 min, vibration frequency at 20 or 25 Hz) improved blood flow in peripheral dorsal foot skin but reduced plantar cutaneous sensation, and older adults showed insufficient improvement in balance and mobility tests [24]. Because little is known regarding the potential effects of using FR or VFR as part of a warm-up protocol on subsequent functional fitness performance in older adults, examining these potentially performance-enhancing effects may be beneficial.

To the best of our knowledge, no prior studies have examined the effects of FR and VFR use on blood pressure and functional fitness performance in older adults. Therefore, the purpose of this study was to examine the acute effects of three warm-up protocols (SS vs. SS combined with nonvibration foam rolling (SS + FR) vs. SS combined with vibration foam rolling (SS + VFR)) on SBP, DBP, HR, MAP, brachial pulse pressure (BPP), senior fitness test (SFT), and single-leg standing balance (SLB) in older women with prehypertension. We hypothesize that compared with SS + FR and SS + VFR, SS may induce greater increases in blood pressure. However, SS + FR and SS + VFR may be more beneficial in the senior fitness test and balance.

## 2. Materials and Methods

### 2.1. Study Design

**Figure 1:**
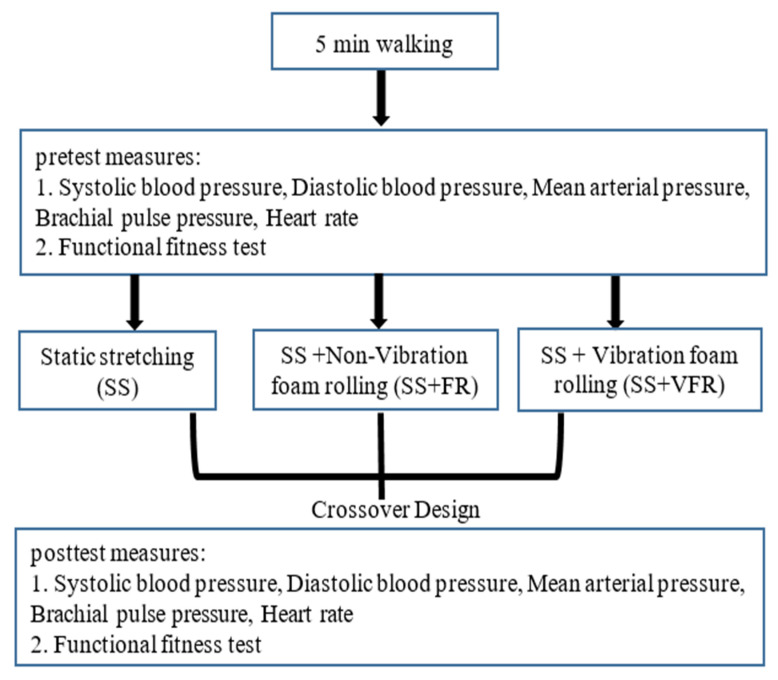
The experimental design of the study.

This study used a within-subject cross-over design to investigate the acute effects of SS, SS + FR, and SS + VFR on SBP, DBP, HR, MAP, BPP, SFT, and SLB. All subjects were familiarized with the experimental procedures a week prior to the first experimental session. In this experiment, three testing sessions were completed by the subjects on three separate days, at least seven days apart. An additional effort was also made to ensure that the tests were conducted at approximately the same time of day for each subject. Vigorous physical activity was not allowed 24 h before each test session. Additionally, any alcohol or caffeine consumption was prohibited a day before the all the experimental visit days. At the beginning of each session, a 5 min walk was performed for the purpose of warming up. Baseline tests consisted of SBP, DBP, HR, MAP, BPP measurements, SFT (back scratch (BS), chair-sit-and-reach (CSR), 30 s arm curl (AC), 30 s chair stand (CS), 2 min step, 8-foot up and go), and SLB tests. After completing pretest measurements, subjects performed SS, SS + FR, or SS + VFR (pre-randomly determined) for that session. Posttest measurements were taken in the same order as pretest measurements immediately after the intervention. A flowchart of the study design is presented in Figure 1.

### 2.2. Subjects

A priori power analysis (G*Power 3.1.9.7, Heinrich-Heine-Universität Düsseldorf, Dusseldorf, Germany) was conducted to determine the sample size [25]. Based on the observed magnitude of the effect from previous studies [23,26], with the alpha level of 0.05 and power (1 − β) of 0.80, a minimum of 12 subjects was required for this experiment. Thirteen elderly women (age: 72 ± 4 years, height: 156.6 ± 5.9 cm, weight: 60.9 ± 9.7 kg, body mass index: 24.79 ± 3.74 kg/m^2^) with untreated prehypertension (SBP/DBP: 120–139/80–89 mm Hg) volunteered to participate in this study. The exclusion criterion includes the presence of any musculoskeletal or cardiovascular diseases. Before the onset of the experiment, all the subjects signed an informed consent form. All experimental procedures were performed in accordance with the Declaration of Helsinki and were approved by the Institutional Review Board of China Medical University Hospital (Approval Number: 109-REC3-107).

### 2.3. Measurements

#### 2.3.1. Blood Pressure and Heart Rate

The pretest and posttest SBP, DBP, and HR were measured with a blood pressure monitor (Omron model HEM-8611, Omron Corporation, Taichung, Taiwan). MAP was calculated as (SBP + [2DBP])/3 [27]. BPP was calculated as the difference between SBP and DBP, with a higher BPP indicating a higher CHD risk [28].

#### 2.3.2. Senior Fitness Test (SFT) and SLB

Physical parameters including shoulder flexibility, lower-body flexibility, lower-body strength, upper-body strength, aerobic endurance, dynamic balance, and static balance [29] were evaluated using the fitness tests BS, CSR, 30 s CS, 30 s AC, 2 min step, 8-foot up and go, and SLB, respectively.

### 2.4. Procedures

During each experimental visit, all subjects underwent one of the following three interventions: SS, SS + VFR, or SS + FR.

#### 2.4.1. Static Stretching (SS)

**Figure 2:**
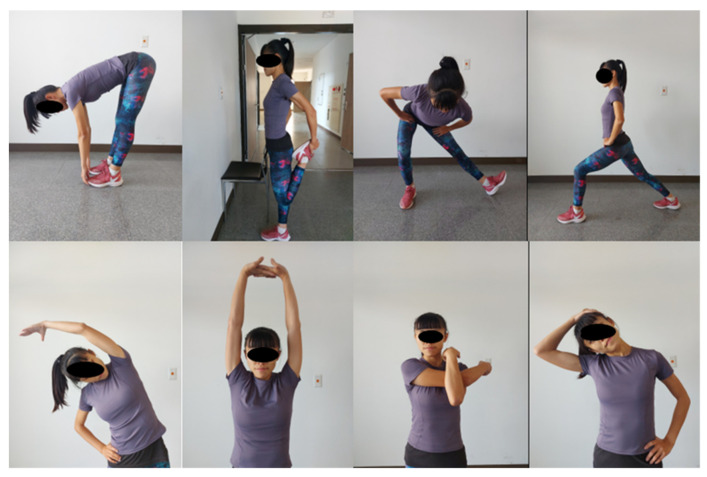
The demonstration of the static stretching (SS) protocol.

Subjects started with a 5 min walk (intensity was maintained at 11 on the 6–20 Borg scale), followed by 14 min of SS and 10 min of rest in a sitting position. The SS involved subjects holding still at the end of the range of motion (ROM) for 30 s for each body part (both sides), and the subjects performed two sets of eight stretching exercises rotationally. The stretching exercises targeted the hamstrings, quadriceps, hip adductors, calves, obliques, upper-back, posterior deltoids, and neck lateral flexor muscles performed to the threshold of mild discomfort without pain (Figure 2).

Top row from left to right: the stretching of hamstrings, quadriceps, hip adductors, and calf muscles. Bottom row from left to right: the stretching of obliques, upper-back muscles, posterior deltoids, and neck lateral flexor muscles.

#### 2.4.2. Static Stretching + Vibration Foam Rolling (SS + VFR)

**Figure 3:**
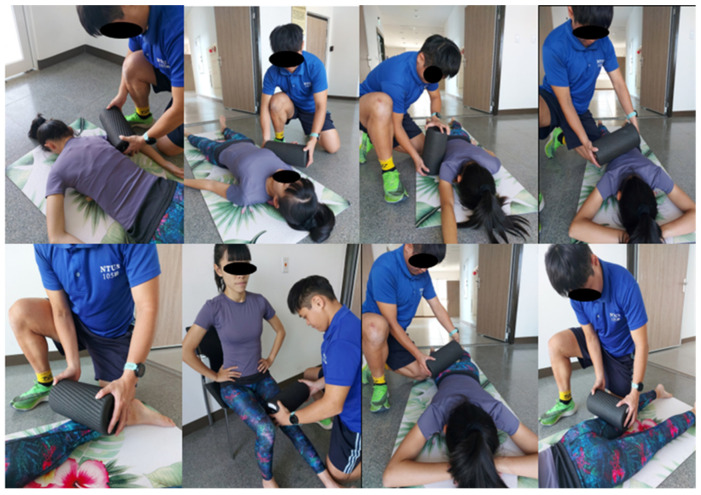
The demonstration of foam rolling (both vibration rolling and nonvibration rolling).

The 5 min walk and one set of 7 min SS were followed by 16 min of passive VFR. For the VFR, a commercial vibration foam roller (Vyper 2.0, Hyperice, Irvine, CA, USA) with a vibration frequency of 48 Hz was used. The VFR was performed passively by a researcher rolling back and forth on the subjects’ triceps brachii, biceps brachii, latissimus dorsi, rotator cuff, calf, quadriceps, gluteus, and hamstring muscles. The pressure applied by the researcher on the subjects’ body parts was maintained at 2 or 3 on a 10 cm visual analog scale based on the subjects’ perception of pressure pain intensity. For each muscle group on each side, one set of VFR was performed for 60 s at a rate of 30 rolls per minute (1 s up and 1 s down) using a metronome (Figure 3). 

Top row from left to right: passive rolling intervention on the triceps brachii, biceps brachii, latissimus dorsi, rotator cuff muscles, calf, quadriceps, gluteus, and hamstring muscles. Bottom row from left to right: passive rolling intervention on the calf, quadriceps, gluteus, and hamstrings muscles.

#### 2.4.3. Static Stretching + Nonvibration Foam Rolling (SS + FR)

The SS + FR was performed with the same methods as those for SS + VFR except without vibration.

### 2.5. Statistical Analyses

Statistical analyses were conducted via SPSS version 19 (Chicago, IL, USA), with all results are reported as the mean ± standard deviation (SD). The Shapiro–Wilk test was performed to examine the normal distribution of the data. Separate (time: pretest vs. posttest) × 3 (condition: SS vs. SS + FR vs. SS + VFR) repeated measures analysis of variance tests were used to analyze the dependent variables. In the case of significant F tests, Bonferroni-adjusted pairwise comparisons were performed to determine significant differences among interventions. Additionally, paired-sample *t*-tests were used to determine differences between pretest and posttest measures during each trial. The effect size (Cohen’s *d*) was reported to represent the magnitude of the effects [30]. Significance for all analyses was set at *p* < 0.05.

## 3. Results

The results for all outcome measures are shown in Table 1 and Table 2. Before tests, no statistical differences for the pre-values were noted among the three warm-up protocols for all dependent variables.

### 3.1. Blood Pressure Parameters

For HR (F = 0.46, *p* > 0.05) and DBP (F = 0.15, *p* > 0.05), the analysis showed no two-way interaction between time and condition and no main effect of condition and time (*p* > 0.05). For SBP, the analysis revealed a two-way interaction between time and condition (F = 4.86, *p* = 0.02). Pairwise comparisons revealed that SBP was significantly lower in the SS + FR group than in the SS (*p* = 0.02) and SS + VFR (*p* = 0.04) groups at the post-time point. Additionally, paired-sample *t*-tests revealed that SBP significantly increased after SS (*p* = 0.003) and SS + VFR (*p* < 0.001). For BPP, the analysis revealed a two-way interaction between time and condition (F = 11.35, *p* < 0.001). Pairwise comparisons revealed that BPP was significantly lower in the SS + FR group than in the SS (*p* < 0.001) and SS + VFR groups (*p* = 0.002) at the post-time point. Additionally, paired-sample *t*-tests revealed that BPP significantly increased after SS and SS + VFR (*p* < 0.001). For MAP, only time had a significant main effect (*p* = 0.04), but no two-way interaction was observed between time and condition (F = 0.09, *p* = 0.91) or main effect for condition (*p* = 0.79).

**Table 1:** Mean ± Standard Deviation of systolic blood pressure, diastolic blood pressure, and heart rate following three warm-up protocols.

	SS			SS + FR			SS + VFR		
	Pre	Post	ES	Pre	Post	ES	Pre	Post	ES
SBP (mmHg)	131.23 ± 14.68	141.31 ± 17.72 *	0.63	131.08 ± 10.29	132.62 ± 20.8 9 ^#^	0.10	131.15 ± 11.22	143.31 ± 13.44 *	0.98
DBP (mmHg)	75.00 ± 9.70	75.62 ± 12.81	0.06	75.15 ± 11.19	76.92 ± 10.90	0.16	75.62 ± 9.64	76.00 ± 11.77	0.04
HR (bpm)	71.84 ± 9.69	72.00 ± 13.47	0.01	73.46 ± 13.11	73.30 ± 13.57	0.01	75.38 ± 9.82	73.85 ± 11.25	0.15
MAP	93.74 ± 10.73	97.51 ± 13.17	0.31	93.23 ± 11.60	96.05 ± 11.72	0.24	93.97 ± 12.01	96.49 ± 13.01	0.20
BPP	56.23 ± 9.33	65.69 ± 13.52 *	0.81	55.92 ± 8.51	55.69 ± 16.52 ^#^	0.02	55.54 ± 11.19	67.31 ± 14.93 *	0.89

SS: Static stretching; FR: Nonvibration foam rolling; VFR: vibration foam rolling; SBP: systolic blood pressure; DBP: diastolic blood pressure; HR: heart rate; MAP: mean arterial pressure; BPP: brachial pulse pressure; ES: effect size of pre-post comparison. * Significant difference compared with pretest (*p* < 0.05). ^#^ Significant difference compared with SS, and compared with SS + VFR (*p* < 0.05).

### 3.2. Fitness Test Performance

The results for the functional fitness test outcomes are presented in Table 2. For CSR (F = 1.18, *p* > 0.05) and 8-foot up and go (F = 0.10, *p* > 0.05), the analysis showed no two-way interaction between time and condition or main effect of condition and time (*p* > 0.05). For BS, the analysis showed a two-way interaction between time and condition (F = 4.17, *p* = 0.03). Pairwise comparisons revealed that BS was significantly higher in the SS + VFR group than in the SS (*p* < 0.001) and SS + FR (*p* = 0.002) groups at the post-time point. The paired-sample *t*-tests revealed that BS performance significantly improved after SS + FR (*p* = 0.04) and SS + VFR (*p* = 0.001). For the 2 min step test, the analysis showed a two-way interaction between time and condition (F = 18.50, *p* < 0.001). Pairwise comparisons revealed that the test results (repetitions) were significantly greater in the SS + FR (*p* = 0.006) and SS + VFR groups (*p* = 0.002) than in the SS group at the post-time point. The paired-sample *t*-tests revealed that 2 min step test results significantly improved after SS + FR and SS + VFR (*p* = 0.003). For the 30 s CS test, only time had a significant main effect (*p* = 0.04), but there was no two-way interaction between time and condition (F = 1.06, *p* = 0.36) or main effect for condition (*p* = 0.70). For the 30 s AC, the analysis revealed a two-way interaction between time and condition (F = 8.31, *p* = 0.002). The pairwise comparisons revealed that scores on the 30 s AC test were significantly higher in the SS + VFR group than in the SS group (*p* = 0.001) at the post-time point. The paired-sample *t*-tests revealed that the 30 s AC repetitions significantly increased after SS + VFR (*p* = 0.001). For SLB, the analysis showed a two-way interaction between time and condition (F = 9.17, *p* = 0.001). Pairwise comparisons revealed that SLB performance (time) was significantly lower in the SS + VFR group than in the SS group (*p* = 0.02) and SS + FR group (*p* = 0.01) at the post-time point. The paired-sample *t*-tests revealed that the SLB time significantly decreased after SS + VFR (*p* = 0.03) but not after SS or SS + FR.

**Table 2:** Mean ± Standard Deviation of upper body flexibility, lower body flexibility, aerobic endurance, lower-body strength, upper-body strength, dynamic balance and agility, and static balance following three warm-up protocols.

	SS			SS + FR			SS + VFR		
	Pre	Post	ES	Pre	Post	ES	Pre	Post	ES
Back scratch (cm)	−1.15 ± 5.77	−0.89 ± 6.22	0.04	−1.58 ± 6.30	−0.64 ± 6.33 *	0.15	−0.92 ± 6.22	0.33 ± 6.01 ^#^*	0.20
Chair sit-and-reach (cm)	4.64 ± 3.78	4.36 ± 5.12	0.06	4.38 ± 5.20	4.88 ± 4.58	0.10	4.74 ± 4.74	5.38 ± 4.27	0.14
2 min step (repetitions)	74.69 ± 22.87	73.62 ± 23.55	0.05	73.30 ± 23.49	76.62 ± 23.68 ^&^*	0.14	74.62 ± 23.44	77.92 ± 25.35 ^&^*	0.14
30 s chair stand (repetitions)	19.85 ± 4.65	20.46 ± 6.37	0.11	19.46 ± 8.20	22.85 ± 9.10	0.39	19.00 ± 5.45	21.15 ± 8.08	0.31
30 s arm curl (repetitions)	26.77 ± 5.70	26.00 ± 5.42	0.14	27.15 ± 8.12	27.85 ± 8.90	0.08	26.54 ± 5.60	30.62 ± 6.01 ^&^*	0.70
8-foot up and go (s)	6.98 ± 1.72	6.85 ± 1.44	0.08	6.63 ± 1.51	6.61 ± 1.54	0.01	6.58 ± 1.52	6.47 ± 1.57	0.07
Single leg stance (s)	13.63 ± 12.09	14.18 ± 12.04	0.05	13.73 ± 11.98	13.98 ± 11.73	0.02	13.91 ± 12.06	13.02 ± 11.57 ^#^*	0.08

SS: Static stretching; FR: Nonvibration foam rolling; VFR: vibration foam rolling. ES: Effect size of pre-post comparison. * Significant difference compared with pretest (*p* < 0.05). ^&^ Significant difference compared with SS (*p* < 0.05). ^#^ Significant difference compared with SS, and compared with SS + FR (*p* < 0.05).

## 4. Discussion

To our knowledge, the current study is one of the few to compare the acute effects of different warm-up protocols (i.e., SS, SS + FR, and SS + VFR) on SBP, DBP, HR, MAP, BPP, SFT, and SLB in older women with prehypertension. The main findings are (1) both SS and SS + VFR warm-up protocols produced significantly greater SBP and BPP compared with those of the SS + FR protocol; (2) the 2 min step test performance after SS + FR and SS + VFR was significantly higher than that after SS; (3) BS performance was significantly improved after SS + VFR compared with after SS and SS + FR; (4) 30 s AC performance significantly improved after SS + VFR compared with after SS; (5) SLB performance was worse after SS + VFR than after SS and SS + FR; and (6) no differential warm-up effects were observed in CSR, 30 s CS, or 8-foot up and go.

Little is known regarding the effects of warm-up protocols such as SS + VFR and SS + FR on blood pressure, and previous study results have been inconsistent. In this study, the SS warm-up protocol significantly increased SBP and BPP. This finding is consistent with many previous reports. In healthy young participants, SS (approximately 5–45 min) can significantly increase blood pressure [12,14,15,16,31]. For example, a single 45 min session of whole-body active SS (4 sets of 20 s) resulted in an acute increase in BPP, SBP, DBP, and MAP in young men [31]. With whole-body SS (3 sets of 30 s) for 40 min, brachial and ankle SBP significantly increased compared with the baseline and 60 min values, and brachial and ankle DBP significantly increased compared with the baseline and 0, 30, and 60 min values [12]. Furthermore, whole-body passive SS for 12 min (3 sets of 20 s) increased SBP and DBP at the midpoint of SS, and lower mean R-R intervals and higher DBP were noted at 10 min after SS [14]. By contrast, only passive calf SS (6 sets of 30 s) showed no change in brachial or ankle SBP, DBP, or HR [32]. These results indicate a higher cardiac overload in the whole-body SS warm-up protocol [14]. In addition, individuals with relatively low flexibility who thus likely require high stretching tension during stretching exercises may experience greater stimulation of the muscle and tendon mechanoreceptors [33,34]. Moreover, SS could possibly result in blood vessel occlusion [35,36], and the possible Valsalva maneuver performed during SS might have also elicited a higher blood pressure response. 

A notable finding of this study is the differential effects of SS + VFR and SS + FR on blood pressure. Specifically, adding the FR component to a typical SS warm-up protocol did not increase the blood pressure. A previous study reported significant decreases in both SBP and DBP after FR [21]. In addition, Lastova et al. [22] observed significant decreases in SBP and DBP at 10 and 30 min after the FR trial. Compression of the muscle arteries by the foam roller triggers the release of plasma nitric oxide and consequently reduces arterial stiffness [23]. Therefore, SS + FR maintains blood pressure response and avoids increased cardiovascular load. However, the current study is the first to show that SS + VFR increases SBP and BPP. Even though the VFR is relatively new and the mechanisms of the VFR on cardiac responses are not understood, this finding is consistent with and may be explained by some previous research examining whole-body vibration (WBV). Figueroa et al. [37] observed significant increases in SBP and HR 5 min after squat with and without WBV (10 sets of 60 s, 40 Hz, 1 mm). In addition, SBP and DBP significantly increased during static squats with WBV (6 sets of 30 s, 35 Hz, 6 mm) and without WBV, and more interestingly, SBP and DBP were higher during exercises with WBV compared with that without vibration in sedentary adults [38]. By contrast, WBV (3 sets of 60 s, 30 Hz, 2 mm) significantly reduced ankle SBP from the baseline value at 30 min, and there were no changes in HR, brachial SBP, brachial or ankle DBP, MBP, or PP [39]. In addition, WBV (3 sets of 30 s, 30 Hz, 3 mm) caused no changes in HR, SBP, DBP, or SpO_2_ in elderly patients with cardiovascular diseases [40]. According to these studies, it seems that higher WBV frequency (>30 Hz) caused greater stimulation of the muscular and cardiovascular systems and contributed to an increase in the overall cardiovascular load [41,42]. Lastly, regarding the blood pressure findings, it is very important to note that it is unclear whether the statistically significant increases in blood pressure (in average, 10–12 mmHg increases for the systolic blood pressure) following SS and SS + VFR were clinically relevant. 

To our knowledge, the present study is the first to explore the acute effects of SS + FR and SS + VFR on functional fitness test results in older women. Our study results indicate that SS + VFR and SS + FR enhance BS (shoulder) flexibility and that SS + VFR is more effective than the other two protocols. Most studies on FR and VFR have focused on the muscle groups around the knee [43,44], while few studies have explored their effects on shoulder flexibility [45]. Compared with other studies, the current study identified a non-significant change of CSR, and this difference may be attributable to the stretching and rolling volume (stretching each muscle for two sets of 30 s and rolling for one set of 60 s). For example, Lee et al. reported that FR, VFR, and SS (3 sets of 30 s for all three warm-up conditions) significantly improved knee extension ROM [44], and FR (3 sets of 30 s) and SS (3 sets of 30 s) were reported to significantly improve CSR results [46]. 

Our results show that SS + VFR is superior to SS in the 2 min step and 30 s AC tests and that SS + FR is more effective than SS in the 2 min step test. Previously, Lee et al. found that FR and VFR significantly increase quadriceps muscle strength and that VFR increases hamstring strength compared with SS [44]. Similarly, Adams et al. advocated whole-body vibration stimulation (range 30–60 s) for its general positive impact on countermovement jump performance [47]. It is important to mention that in some previous studies [44,46,48] participants performed FR or VFR on the floor by actively rolling back and forth on each muscle while maintaining body stability and balance. In the current study, to ensure the safety of the older adults, passive rolling was performed and the core muscle group was not used, which might have affected the participants’ lower limb strength performance (e.g., 30 s chair stand).

Regarding the effects on SLB, we found that participants had significantly poorer SLB performance after SS + VFR and no change after SS and SS + FR. Thus, the vibration component of the SS + VFR seemed to impose a negative effect on the participants’ balance. Previously, Pollock et al. employed WBV (5 sets of 60 s and 30 Hz) with two amplitudes (4 and 8 mm) and reported decreased cutaneous sensation at the foot, ankle, and posterior shank immediately and until 30 min after high-amplitude WBV [49], which may impair proprioceptive feedback and balance in young healthy participants or older adults with high fall risk. Therefore, these situations should be considered during the application of SS + VFR for individuals with sensory impairment and those with a greater fall risk, especially older adults. 

This study has some limitations. First, the findings of the present investigation cannot be extrapolated to other populations (e.g., young and older men). Second, the rolling intervention in the current study was investigated under three conditions: passive, relatively high-velocity, and with one fixed frequency. It is important to note that all the rolling interventions were performed by the professionals, and therefore, the current findings may not be used to speculate other rolling interventions such as self-administered foam rolling. Third, we only examined the variables immediately after the warm-up interventions, and we are not clear how long these effects can last. Thus, cautions need to be taken when interpreting our findings, and future research is warranted to further examine the potential time course of the warm-up effects. 

## 5. Conclusions

In conclusion, although SS + VFR was superior to SS in the BS, 2 min step, and 30 s AC tests, it could impair SLB performance. In addition, SS + VFR increased SBP and BPP, which indicates a greater risk of adverse cardiovascular events in older adults. Practically speaking, therefore, older women who are prehypertension or have other cardiovascular risks may benefit more from performing a static stretching with foam rolling warm-up, because this warm-up intervention does not add an acute stress to the cardiovascular system, while it can still serve as an effective warm-up protocol.

## Data Availability

The data presented in this study are available on request from the corresponding author.
